# Antibiotics for paediatric community-acquired pneumonia in resource-constrained settings

**DOI:** 10.1183/13993003.02773-2020

**Published:** 2020-09-17

**Authors:** Amy Sarah Ginsburg, Keith P. Klugman

**Affiliations:** 1 University of Washington, Seattle, WA, USA; 2 Bill and Melinda Gates Foundation, Seattle, WA, USA

## Abstract

Despite *Streptococcus pneumoniae* and *Haemophilus influenzae* type b vaccination strategies, pneumonia remains the leading infectious cause of child mortality. Greater access to appropriate treatment is critical; however, defining “appropriate” is problematic. World Health Organization (WHO) guidelines recommend diagnosing pneumonia using clinical signs and a non-specific, pragmatic case definition: fast breathing or chest indrawing (pneumonia) and presence of WHO danger signs (severe pneumonia) in children with cough or difficulty breathing [1]. It is unclear whether all “pneumonia” using these definitions needs to be treated with antibiotics, and if so, for how long.

## Abstract 2

**3 days of amoxicillin may be sufficient to treat most non-severe community-acquired paediatric pneumonia in resource-constrained settings** https://bit.ly/3jmQSTX

*To the Editor:*

Despite *Streptococcus pneumoniae* and *Haemophilus influenzae* type b vaccination strategies, pneumonia remains the leading infectious cause of child mortality. Greater access to appropriate treatment is critical; however, defining “appropriate” is problematic. World Health Organization (WHO) guidelines recommend diagnosing pneumonia using clinical signs and a non-specific, pragmatic case definition: fast breathing or chest indrawing (pneumonia) and presence of WHO danger signs (severe pneumonia) in children with cough or difficulty breathing [1]. It is unclear whether all “pneumonia” using these definitions needs to be treated with antibiotics, and if so, for how long.

Implementation of WHO guidelines, designed to deliver improved quality of care despite limited resources, has contributed to marked reductions in child mortality. Yet, WHO criteria for pneumonia have low specificity, resulting in misclassification [2]. With a changing epidemiological context, reassessment of the necessity and duration of antibiotics is warranted. Overtreatment with antibiotics of children with cough and fast breathing may have unintended, individual and public health consequences by contributing to emergence of antimicrobial resistance [3]. Exposing children to longer antibiotic courses, their adverse events, and unintended collateral damage on symbiotic bacteria in the child's forming microbiome and immunity should be avoided [4]. Understanding optimal antibiotic duration is required to treat pneumonia effectively, while circumventing unnecessarily prolonged courses that have negative adherence, tolerance and cost implications.

Two double-blind, randomised controlled non-inferiority trials of WHO fast-breathing pneumonia were conducted to evaluate whether treatment with placebo in HIV-uninfected children aged 2 to 59 months is as effective as 3-day oral high-dose amoxicillin, the current WHO-recommended treatment [5, 6]. Both trials found placebo significantly inferior to amoxicillin (Malawi: 7.0% (38/543) *versus* 4.0% (22/552); difference 3.0% (95% CI 0.4–5.7%); Pakistan: 4.9% (995/1927) *versus* 2.6% (51/1929); difference 2.3% (95% CI 0.9–3.7%)). However, no treatment failure by day 4 was observed in ∼93% and ∼95% of children receiving placebo in Lilongwe, Malawi and Karachi, Pakistan, respectively. There was no significant difference in treatment failure or relapse by day 14. In Malawi, the number needed to treat with amoxicillin for one child to benefit was 33 and in Pakistan, 44.

The vast majority of children did not require antibiotics and the fraction of true bacterial pneumonia was likely low. WHO pneumonia is a syndromic classification, and without microbiological diagnosis, aetiological ascertainment was not possible. Given the broad definition of treatment failure and variability in trajectory of illness, some treatment failure cases were likely persistent viral infections, needing more time to resolve. With viruses increasingly aetiological agents [7], further research is needed to define which children actually benefit from antibiotics within this clinical definition.

If treatment is warranted, it is also unclear for how long [8]. Given 6.4% relapse *versus* 4.0% treatment failure rates in Malawi and 3.1% relapse *versus* 2.6% treatment failure rates in Pakistan among children with fast-breathing pneumonia who received 3-day amoxicillin, it is worth considering whether a longer course of amoxicillin was required [5, 6]. A double-blind, randomised controlled non-inferiority trial conducted in Lilongwe, Malawi found three-day oral high-dose amoxicillin in HIV-uninfected children aged 2 to 59 months with WHO chest-indrawing pneumonia, non-inferior to 5-day amoxicillin, the current WHO-recommended treatment [9]. Children receiving 3-day amoxicillin had a 5.9% (85/1442) treatment failure rate by day 6, within the non-inferiority margin of those receiving 5-day amoxicillin (5.2% (75/1456) treatment failure rate). No significant difference between 3-day and 5-day amoxicillin in treatment failure or relapse was observed by day 14. Serious adverse events were similar between groups (9.8% for 3-day *versus* 8.8% for 5-day). If 3-day amoxicillin is sufficient to treat chest-indrawing pneumonia, then 3-day amoxicillin is likely sufficient to treat fast-breathing pneumonia.

There are individual and health system benefits of shorter course treatment. Poor adherence is associated with treatment failure in WHO-defined clinical pneumonia [10]. Improving adherence with shorter course antibiotic treatment could improve outcomes in children with chest-indrawing pneumonia while also minimising adverse drug effects, costs and antimicrobial resistance [8, 10]. Given that WHO recommends 3-day amoxicillin for fast-breathing pneumonia [1], 3-day amoxicillin for chest-indrawing pneumonia would enable harmonisation of treatment for non-severe pneumonia among HIV-uninfected children.

Existing pneumonia management guidelines maximise sensitivity over specificity and can lead to widespread over-prescription of antibiotics and antibiotic resistance. While the majority of fast-breathing pneumonia may not be bacterial requiring antibiotics, some invariably do. In the absence of a rapid, accurate, point-of-care test to definitively diagnose pneumonia and further differentiate bacterial *versus* viral aetiology, frequent, comprehensive follow-up assessments with improved objective criteria are required to identify the small subset benefiting from antibiotics. Moving away from only a clinically based definition of pneumonia to include diagnostics such as lung ultrasound to help predict disease course and inform management may provide clarity regarding which children require treatment with antibiotics and for how long.

## Shareable PDF

10.1183/13993003.02773-2020.Shareable1 This one-page PDF can be shared freely online.
Shareable PDF ERJ-02773-2020.Shareable
