# Supplementary material for: Antibiotics for paediatric community-acquired pneumonia in resource-constrained settings
Source: Eur Respir J. 2020 Sep 17;56(3):2002773. doi: 10.1183/13993003.02773-2020 (PMC7494842; doi:10.1183/13993003.02773-2020)
Supplement: Supplementary file 1 [file ERJ-02773-2020.Shareable.pdf]

SHAREABLE PDF

Antibiotics for paediatric community-acquired pneumonia in resource-constrained settings

Amy Sarah Ginsburg[1] and Keith P. Klugman[2]

Affiliations: [1]University of Washington, Seattle, WA, USA. [2]Bill and Melinda Gates Foundation, Seattle, WA, USA.

Correspondence: Amy Sarah Ginsburg, University of Washington Clinical Trial Center Building 29, Suite 250, 6200 NE 74th Street, Seattle, WA, 98115, USA. E-mail: messageforamy@gmail.com

@ERSpublications
3 days of amoxicillin may be sufficient to treat most non-severe community-acquired paediatric pneumonia in resource-constrained settings https://bit.ly/3jmQSTX

Cite this article as: Ginsburg AS, Klugman KP. Antibiotics for paediatric community-acquired pneumonia in resource-constrained settings. *Eur Respir J* 2020; 56: 2002773 [https://doi.org/10.1183/13993003.02773-2020].

This single-page version can be shared freely online.

To the Editor:

Despite *Streptococcus pneumoniae* and *Haemophilus influenzae* type b vaccination strategies, pneumonia remains the leading infectious cause of child mortality. Greater access to appropriate treatment is critical; however, defining "appropriate" is problematic. World Health Organization (WHO) guidelines recommend diagnosing pneumonia using clinical signs and a non-specific, pragmatic case definition: fast breathing or chest indrawing (pneumonia) and presence of WHO danger signs (severe pneumonia) in children with cough or difficulty breathing [1]. It is unclear whether all "pneumonia" using these definitions needs to be treated with antibiotics, and if so, for how long.

Copyright ©ERS 2020.. This version is distributed under the terms of the Creative Commons Attribution Licence 4.0.
